# Pediatric herpes zoster: should I be concerned for immunodeficiency? A review

**DOI:** 10.3389/fped.2025.1561339

**Published:** 2025-03-14

**Authors:** Steven Zhang, Vy H. D. Kim, Eyal Grunebaum

**Affiliations:** Division of Immunology and Allergy, Department of Pediatrics, The Hospital for Sick Children, Toronto, ON, Canada

**Keywords:** herpes zoster, varicella-zoster virus, pediatrics, immunodeficiencies, inborn errors of immunity

## Abstract

Herpes zoster (HZ), caused by reactivation of varicella-zoster virus (VZV), is an uncommon cause of rash in pediatrics, which can lead to concerns of an underlying immunodeficiency. We reviewed studies on HZ in pediatric patients. The diagnosis of HZ can usually be established based on clinical and epidemiological features. HZ is associated with T-cell immune defects that can be secondary to infections with HIV, tuberculosis, and other pathogens, as well as conditions such as diabetes, malnutrition, cancer, or primary immunodeficiency. Important clinical clues indicating that HZ is due to an underlying immunodeficiency include recurrent HZ during a short period; disseminated HZ; new lesions more than a week after presentation; prolonged course despite antiviral medications; a history of recurrent, invasive, or prolonged infections by other pathogens; and a family history of immunodeficiency or consanguinity. Reassuring features include exposure to VZV prior to 1 year of age or a compromised or incomplete VZV vaccination schedule. Initial laboratory analysis may include confirmation of normal newborn screening for profound T-cell immunodeficiency; a complete blood count with differential, quantitative serum immunoglobulins; lymphocyte subset analysis; and the presence of IgG to VZV. In children previously vaccinated for VZV, the possibility of vaccine-type HZ needs to be considered. In conclusion, isolated and uncomplicated childhood HZ is unlikely to be the sole harbinger of an underlying immunodeficiency. Therefore, most children with HZ can be adequately diagnosed through medical history and readily available laboratory evaluations. The presence of concerning clinical or laboratory features should prompt an evaluation by an experienced specialist.

## Introduction

Primary infection with varicella-zoster virus (VZV) causes varicella, commonly known as “chicken pox,” which is characterized by disseminated scattered maculopapular vesicular rash and fever. In healthy children, chicken pox is generally a mild and self-limited disease, although complications such as dehydration, secondary bacterial infections, and Reye syndrome can occur in 2%–10% of cases (1). Following a primary infection with VZV or vaccination with a live attenuated VZV, the virus migrates to the dorsal or cranial nerve ganglia, where it remains latent for many years. Reactivation and subsequent replication of VZV results in disease of the ganglion, the nerve, and the innervated skin known as herpes zoster (HZ) or shingles. HZ typically manifests as unilateral radicular pain with a clustered, maculopapular rash that may become vesicular and is often limited to a single dermatome (2). HZ is common in older individuals, with an incidence rate of 3–5/1,000 person-years and an even higher incidence after 50 years of age, including approximately 5% reoccurrence (3). In contrast, HZ is an uncommon cause of pediatric rash, and its identification in children can lead to concerns about immune incompetence. Here, we review the clinical presentation and diagnosis of HZ in children, describe the pathophysiology of HZ, and detail clinical and laboratory features that can help alleviate or substantiate concerns that HZ is an indicator of an underlying immunodeficiency.

## Methods

We reviewed the publicly accessible PubMed, OVID, and Scopus bibliographic databases for English-language studies on HZ in children using the search terms varicella-zoster virus, VZV, herpes zoster, shingles, recurrent, children, pediatrics, and immunodeficiency, without limitation of the search dates. The search was conducted between August and October 2024. Full-length manuscripts that contained the search terms in their title or abstract were further explored for relevance to the scope of the current review.

### Clinical presentation and diagnosis of herpes zoster in children

The clinical presentation of HZ in children is often similar to those in older individuals, with a single dermatome rash that typically has a 1-week course, ultimately becoming pustular before ulcerating and crusting (4). Adjacent dermatomes are infrequently affected, with vesicles scattering outside the primary eruption, and rarely few distant lesions can develop, possibly due to an early blood viremia. Some features have been identified in pediatric HZ that differ from the adult disease (Table 1). Thoracic dermatomes are more commonly affected in children, while in adults cranial nerve ganglions are frequently involved (5). Additionally, pediatric HZ is often pruritic rather than the painful adult HZ. Pediatric HZ can also be associated with systemic symptoms of low-grade fever, malaise, and headache (6). As for complications, streptococcal and staphylococcal bacterial superinfection of the skin lesions is most common in children, often due to *Streptococcus* pyogenes, group A (7, 8), while severe complications such as sepsis and meningitis or postherpetic neuralgia are rare in children compared with adults (9).

**Table 1:** Features distinguishing pediatric and adult herpes zoster.

	Pediatric	Adult
Rash	Generally mild, associated with pruritus. Rash in the lumbosacral area suggests a vaccine-related HZ	Variable severity, associated with pain (can be debilitating)
Illness severity and duration	Shorter, less frequent hospitalization	Longer, more frequent hospitalization
Complications	Infrequent	Frequent
	Most common: secondary bacterial skin infection	Most common: postherpetic neuralgia

It is important to distinguish between HZ and reoccurrence of chicken pox, which has been reported in healthy children (10), including those who were vaccinated, particularly if vaccination was incomplete. Recurrent chicken pox has also been identified among consecutive generations in otherwise healthy members of families (10). Several factors help distinguish recurrent chicken pox from HZ (Table 2). These factors include the age of the patient, the limited dermatome distribution of the lesions in HZ, the prodrome, and the painful rash in HZ vs. the intense itch accompanying chicken pox. Cutaneous lesions of HZ also need to be differentiated from herpes simplex, dermatitis herpetiformis, impetigo, contact dermatitis, candidiasis, drug reactions, and insect bites (11).

**Table 2:** Features distinguishing recurrence of primary varicella infection from herpes zoster.

	Recurrence of primary VZV infection	Reactivation of latent VZV infection (herpes zoster)
Distribution	Generalized, bilateral rash with lesions in various stages of development	Unilateral dermatomal rash with lesions that progress uniformly
Prodrome	Fever and malaise	Localized pain, pruritus, or paresthesia in affected dermatome
Age	Common in children	Common in adults over 50

Clinical and epidemiological features usually are sufficient for the diagnosis of HZ with no need for additional testing. VZV serology can be considered in patients with unknown history of chicken pox or VZV vaccination, although depending on the timing of the testing, this will not conclusively establish or exclude the diagnosis. In inconclusive or atypical cases of children previously vaccinated with the VZV vaccine, demonstration of Oka strain VZV DNA in the vesicular fluid by polymerase chain reaction, performed in several references labs, can help confirm the diagnosis. Direct immunofluorescence is another option but is less sensitive than the polymerase chain reaction method (4).

### Pathophysiology: the immune response to VZV

Host innate and adaptive immune responses are essential to limit VZV disease and prevent reactivation (12). During primary infection, the innate immune system pattern recognition receptors sense the VZV molecular signature. Toll-like and intracellular receptors recognize VZV particles and DNA, which leads to the production and release of proinflammatory cytokines such as interferon that in turn inhibit viral replication and recruit diverse inflammatory cells, including plasmacytoid dendritic cells and natural killer cells (12). IgM, followed by IgG antibodies to VZV, plays an important role in controlling primary VZV infection and reactivation, while VZV latency is largely governed by T-cell-mediated immunity. Within 2 weeks of VZV reactivation, VZV-specific CD4 T cells with a terminal effector phenotype can be detected, which decline after 3–6 weeks (12). Given the central role of T cells in prevention of HZ, it is not surprising that HZ has been associated with many secondary (Supplementary Table 1) and primary (Supplementary Table 2) T-cell defects. HIV and T-cell immune suppressive/modulatory medications are common causes of HZ. Immune senescence; mental stress; infections with tuberculosis, cytomegalovirus, Epstein–Barr virus, or SARS-CoV-2 coronavirus; diabetes; malnutrition; and cancer, which directly or indirectly compromise T-cell function, can also contribute to the development of HZ. Severe VZV and HZ, including recurrent episodes, have been reported with diverse inborn errors of immunity. Infants with the most profound T-cell defects, known as “severe combined immunodeficiency” (SCID), can suffer in the first few years of life disseminated and fatal chicken pox or HZ following VZV primary infection or vaccination or VZV reactivation (13). Typically, patients with SCID have very low or absent T cells enabling their identification by reduced T-cell receptor excision circles in newborn screening programs that have been initiated in the USA, Canada, Europe, and other countries in recent years. Moreover, patients with SCID are susceptible to other infections; hence, most will present prior to the development of HZ. However, some immune abnormalities that are less severe, such as the “combined immune deficiencies,” where T-cell numbers and functions are only moderately compromised, or immune defects involving interferon signaling or natural killer cells, might present with HZ beyond the first years of life (14–16). The concerns that HZ, and particularly recurrent HZ, in children might be due to an underlying immune dysfunction, appearing prior to the diagnosis of the underlying immunodeficiency, has led some health care providers to perform extensive investigations of the immune system. In the following sections, “red flags” for immunodeficiency vs. reassuring features for immune competence, and potential evaluations that may be considered, will be discussed.

### Should I be concerned that HZ is due to an immunodeficiency? “Red flags” for an immune defect

In most pediatric patients presenting with HZ, a comprehensive history can identify “red flag” features concerning an underlying immunodeficiency, such as a history of recurrent, invasive, or prolonged infections. Various sets of warning signs for immunodeficiency have been established, such as those published by the Jeffrey Modell Foundation (17) and the Dusseldorf criteria (18). In addition to frequency, these criteria emphasize the severity of the infection and the need for intravenous antibiotics. Furthermore, developing severe infections from cytomegalovirus, Epstein–Barr virus, candida, or atypical mycobacteria as well as *Pneumocystis jirovecii* pneumonia can indicate immune incompetence (19). Failure to thrive is another symptom of a potential underlying immunodeficiency due to poor nutrient absorption or increased metabolic demand (20). Although less specific, cutaneous manifestations, such as widespread or difficult-to-treat eczema, granulomas, or dysplasia of the skin, hair, and nails, can be found in primary immunodeficiency disorders (21). Persistent lymphadenopathy and splenomegaly or inappropriately rudimentary lymph nodes and tonsils are also concerning. An important non-infectious manifestation is recurrent, persistent, or difficult-to-treat autoimmune cytopenia (22). While most cases of autoimmune cytopenia self-resolve or respond to first-line treatment with corticosteroids or intravenous immunoglobulin, patients with underlying immunodeficiency often require second- and third-line therapy (23). Another concerning feature would be symptomatic infections following vaccination with live attenuated pathogens, such as rotavirus, measles, mumps, rubella, VZV, or the *Bacillus* Calmette–Guérin vaccine (24). Other key “red flags” of immunodeficiency include a family history of recurrent unexplained infections, autoimmunity, or atypical malignancies (25). In countries and communities with high rates of consanguinity, autosomal recessive primary immunodeficiencies occur at higher prevalence (26). Finally, laboratory features such as unexplained neutropenia, lymphopenia, thrombocytopenia, or anemia should raise the possibility of an underlying immunodeficiency.

### Should I be concerned that HZ is due to an immunodeficiency? Reassuring features

In the absence of “red flags,” an isolated presentation of uncomplicated and short HZ in a pediatric patient is unlikely to represent an underlying immunodeficiency. The vast majority of pediatric HZ resolve completely with no long-term sequelae (27). Although immunocompromised children have a 5–6 times higher risk of HZ, HZ is rarely the presenting feature of immunodeficiency (28). Long-term studies have not demonstrated isolated childhood HZ as the sole harbinger of an underlying immunodeficiency, HIV infection, or malignancy (6, 27). Hence, while malignancies, including leukemia and Hodgkin's lymphoma, are frequently associated with pediatric HZ, this is with relapsed disease or with chemotherapy- or radiotherapy-induced immunosuppression rather than the initial presentation (29).

In immunocompetent children, a primary VZV infection during the first year of life is associated with a higher incidence of subsequent HZ (4, 30), with an increased relative risk ranging between 2.8 and 20.9 (6). The occurrence of HZ following VZV at an early age, including intrauterine exposure, has been attributed to a lower-than-normal development of cellular and humoral immunity to VZV, and as such, would not be concerning for an underlying immunodeficiency (31, 32). HZ can also develop during or following infection by other pathogens that temporarily suppress the immune system, such as COVID-19. For example, a 20-month-old patient suffered from progressive VZV vaccine strain HZ 4 days after testing positive for SARS-CoV-2 (33). Other reassuring features in a pediatric HZ presentation include improper storage or administration of the vaccine and non-adherence to the recommended VZV vaccination schedule, including administration of the vaccine in the first year of life (28). Moreover, while VZV vaccines significantly reduced the risk of subsequent HZ in adults (34), there are conflicting data on the effects of the VZV vaccine on the frequency of subsequent HZ in children. Before the introduction of routine VZV vaccination, HZ incidence rates among children and adolescents worldwide ranged from 0.2 to 2.2 per 1,000 person-years, depending in part on the age of the children (6). In the USA, during a period with high varicella vaccine coverage, there was a 72% decrease in overall HZ incidence to 0.2–0.4 per 1,000 person-year in children <18 years of age (35). Yet, other studies found that the incidence of HZ plateaued with the introduction of the VZV vaccine (36) or even increased, particularly among older children (37).

### Should I be concerned that HZ is due to the reactivation of the varicella vaccine virus?

In countries where the VZV vaccine is widely used, severe HZ is commonly caused by the vaccine virus, often with meningitis or meningoencephalitis. All of these cases occurred in children, most of whom were immunocompetent. The reported cases occurred in the USA, the UK, Germany, Switzerland, and Japan (38). All varicella isolates were determined to be vaccine-type by reputable labs (39). The cases occurred in children who had been given one or two doses of the vaccine. If the child had had two doses of the vaccine, the HZ event tended to occur in adolescence. An important clue to the cause of HZ is the localization of HZ. Since the first vaccine dose is given in the thigh, the virus usually travels to the adjacent lumbosacral dorsal root ganglia to establish latency (39). Subsequent HZ then spreads to lumbosacral dermatomes. In contrast, after wild-type infection, lumbosacral dermatomes would be an uncommon location. Hence, if a child appears with a lumbosacral dermatomal HZ, it is likely caused by a vaccine-type virus.

### Should I be concerned that HZ is due to an immunodeficiency? Differences in the HZ course

The course of the patient's HZ infection itself can provide important insight into the individual's immune status. The presence of prolonged fever; the involvement of sacral dermatomes (5); a prolonged course, particularly if this occurs despite treatment with antiviral medications; the development of new lesions more than a week after presentation (40); or invasive HZ leading to pneumonia, hepatitis, retinitis, or meningoencephalitis (14, 25, 41) should raise concerns for a possible underlying immunodeficiency. Similarly, HZ involving multiple dermatomes has been reported in up to 40% of immunocompromised patients while less likely to occur in otherwise healthy children (42, 43). Complications in the immunocompetent population are common during the early stages of disease and involve superinfection of the skin and eyes (42, 44). In contrast, postherpetic neuralgia and facial palsy are more common complications in immunocompromised patients (42). There are conflicting reports on whether complications of HZ are less common and severe in immunocompetent than in immunocompromised children when prompt antiviral treatment is provided (7, 42). Recurrent HZ, which is reported to occur in <2% of children (45), has also been considered a potential indicator of immunodeficiency, particularly if the intervals between the HZ episodes are within a few weeks or months. In contrast, reoccurrence of HZ many years apart, particularly if there are mitigating factors and no other indicators of immune abnormalities, is unlikely to represent a significant immunodeficiency.

### Should I be concerned that HZ is due to an immunodeficiency? Initial work-up

Clinicians who are concerned for an underlying immunodeficiency in a pediatric patient with HZ can often initiate first-line investigations of the immune system while using normal reference values adjusted to the age of the child. These could include the following:

- Confirmation of normal newborn screening for SCID from local health authorities, if done
- Complete blood count with differential
- Quantitative serum IgG, IgA, and IgM
- CD4+ and CD8+ T-lymphocyte subset analysis
- IgG to VZV

In a child with HZ rash and previous history of VZV vaccine, demonstration of vaccine strain DNA in the vesicular fluid, which can be done in a growing number of reputable microbiology labs, is another investigation that can offer reassurance in unclear cases.

Combined HIV antigen and antibody testing can be considered in regions with a higher incidence of HIV or specific concerns. Although HZ is not generally the initial manifestation of HIV infection in children from Western countries, studies in India and Central Africa have reported new HIV diagnoses after hospitalization for HZ (46, 47).

The decision to investigate for potential malignancy should be made on a case-by-case basis. Prognostic studies of pediatric HZ patients have not shown a correlation between HZ and the risk of malignancy (45). However, very rare cases have been reported. Among 173 children with HZ in Rochester, MN, USA, one was later diagnosed with lymphoma (6). Based on clinical history and physical examination, work-up can include imaging (chest radiograph, abdominal ultrasonography) and laboratory investigations (complete blood count, erythrocyte sedimentation rate, C-reactive protein, liver and kidney function tests, uric acid, lactate dehydrogenase, and electrolytes).

Clinical and or laboratory “red flags” for an underlying immune defect or inability to properly investigate and interpret concerns for a primary or secondary immunodeficiency should lead to a referral for a more thorough evaluation by an experienced healthcare specialist.

## Conclusion

Pediatric HZ is a reactivation of VZV that can occur in both immunocompetent and immunocompromised children. An isolated and uncomplicated childhood HZ is unlikely to be the sole harbinger of an underlying pediatric immunodeficiency, and an extensive work-up is not routinely indicated. In contrast, the presence of clinical or laboratory red flags for immunodeficiency, including disseminated HZ with generalized cutaneous involvement, severe systemic complications, or repeated HZ, warrants further evaluation by an experienced specialist.
