# Supplementary material for: Pediatric herpes zoster: should I be concerned for immunodeficiency? A review
Source: Front Pediatr. 2025 Mar 14;13:1561339. doi: 10.3389/fped.2025.1561339 (PMC11949924; doi:10.3389/fped.2025.1561339)
Supplement: Supplementary file 1 [file Supplementaryfile1.pdf]

Supplemental Table 1. Secondary defects of T-cell immunity associated with herpes zoster

Aging	Diabetes
Mental stress	Malnutrition
Human immunodeficiency virus	Malignancy
Tuberculosis	Rheumatological diseases
Cytomegalovirus,	Immune suppressive medications
Epstein Barr virus	Hematopoietic cell transplant
SARS-CoV-2	Organ transplant

Supplemental Table 2. Primary defects of T-cell immunity associated with herpes zoster

Activated PI3K-δ syndrome 1	Purine nucleoside phosphorylase deficiency
Adenosine deaminase deficiency delayed/late onset	RMRP deficiency
Autosomal dominant STAT1 gain-of-function	STAT5B deficiency
Autosomal dominant STAT3 deficiency (hyperIgE syndrome)	STK4 deficiency
DOCK8 deficiency	TAPBP deficiency
iNKT cell deficiency	TLR3 deficiency
MAGT1 deficiency	Uncoordinated 119 deficiency
MST1 deficiency	

This file has been converted from its original format for security purposes. Please use CF38F56A21CD1 as a reference.
